# Nucleoside Reverse Transcriptase Inhibitor (NRTI)-Induced Neuropathy and Mitochondrial Toxicity: Limitations of the Poly-γ Hypothesis and the Potential Roles of Autophagy and Drug Transport

**DOI:** 10.3390/pharmaceutics16121592

**Published:** 2024-12-13

**Authors:** John Haynes, Arnav Joshi, Ross C. Larue, Eric D. Eisenmann, Rajgopal Govindarajan

**Affiliations:** 1 Division of Pharmaceutics and Pharmacology, College of Pharmacy, The Ohio State University, Columbus, OH 43210, USA; haynes.343@buckeyemail.osu.edu (J.H.); joshi.454@buckeyemail.osu.edu (A.J.); eisenmann.11@osu.edu (E.D.E.); 2 Department of Cancer Biology and Genetics, College of Medicine, The Ohio State University, Columbus, OH 43210, USA; larue.22@osu.edu; 3 Translational Therapeutics, The Ohio State University Comprehensive Cancer Center, Columbus, OH 43210, USA

**Keywords:** NRTI, antiretroviral drugs, antiretroviral toxic neuropathy, HIV-induced neuropathy, peripheral neuropathy

## Abstract

Nucleoside reverse transcriptase inhibitors (NRTIs) are the backbone of highly active antiretroviral therapy (HAART)—the current standard of care for treating human immunodeficiency virus (HIV) infection. Despite their efficacy, NRTIs cause numerous treatment-limiting adverse effects, including a distinct peripheral neuropathy, called antiretroviral toxic neuropathy (ATN). ATN primarily affects the extremities with shock-like tingling pain, a pins-and-needles prickling sensation, and numbness. Despite its negative impact on patient quality of life, ATN remains poorly understood, which limits treatment options and potential interventions for people living with HIV (PLWH). Elucidating the underlying pathophysiology of NRTI-induced ATN will facilitate the development of effective treatment strategies and improved patient outcomes. In this article, we will comprehensively review ATN in the setting of NRTI treatment for HIV infection.

## 1. Introduction

Human Immunodeficiency Virus (HIV-1), the causative agent of Acquired Immunodeficiency Syndrome (AIDS), is an infectious disease transmitted through bodily fluids, with significant morbidity and mortality worldwide [1]. Since HIV was recognized as an epidemic in the mid-1980s, an estimated ~88.4 million people have been infected with HIV, and ~42.3 million people have died of an AIDS-related illness [2]. Because HIV-1 is a retrovirus, antiretroviral drugs have proved highly effective in the treatment of the disease [1]. The current standard treatment strategy, highly active antiretroviral therapy (HAART), is a combination regimen of multiple antiretroviral drugs [3]. HAART consists of a “backbone” of two nucleoside reverse transcriptase inhibitors (NRTIs) plus one non-nucleoside reverse transcriptase inhibitor (NNRTI), protease inhibitor (PI), or integrase inhibitors (INST) [3]. For instance, Atripla—a coformulation of Efavirenz/Emtricitabine/Tenofovir. It is worth mentioning here that tenofovir is technically a nucleotide analog, but we will group tenofovir with the NRTIs for the sake of this review.

Antiretroviral drug therapies have improved the lives of people living with HIV (PLWH). However, chronic exposure to these therapies may cause toxicities. Of particular interest is peripheral neuropathy caused by NRTIs. Since NRTIs are important for the current standard of care regimens, it is crucial to understand the underlying mechanisms of ATN to mitigate nerve toxicities and improve patient adherence. This review focuses on NRTIs and discusses mechanisms of mitochondrial dysfunction and the role of drug transporters in mediating NRTI-induced neuropathy.

## 2. Nucleoside Reverse Transcriptase Inhibitors (NRTIs)

Despite their important role in checking HIV infection, chronic exposure to NRTIs causes treatment-limiting toxic effects [4]. Table 1 summarizes the adverse effects associated with each of the NRTIs. One of the most common [5] yet poorly understood toxicities caused by NRTI is peripheral neuropathy [6]. Of people living with AIDS, 30% to 60% are affected by peripheral neuropathy [7]. While HIV itself can cause neuropathy (HIV-related sensory neuropathy (HIV-SN)), manifesting as a distal sensory polyneuropathy [8,9], there is evidence that NRTIs themselves may cause neuropathy (antiretroviral toxic neuropathy; ATN). ATN is a common therapy-induced toxicity affecting 11–66% of patients taking NRTIs [10]. These ATN incidence rates vary among the NRTI monotherapies—greater than 30% with zalcitabine (ddC), 23–30% with didanosine (ddI), and 31% with Stavudine (d4T) [11,12]. ATN affects the distal areas, including hands and feet, with painful dysesthesia, a burning sensation, and sensory loss [13,14].

Among all the antiretroviral agents, NRTIs have a greater potential for toxicity to peripheral nerves [13]. The frequency of ATN also increases with higher doses of these NRTIs as well as in combination drug therapy [15]. Severe toxicity and intolerability are at least in part why these drugs were discontinued or withdrawn from the US market. However, NRTI (such as d4T) prescription remains common in low-income countries like those in Africa [16,17]. Although currently available NRTIs, including zidovudine (AZT), lamivudine (3TC), emtricitabine (FTC), abacavir (ABC), and tenofovir (TDF), are less strongly associated with peripheral neuropathy, ATN remains a relevant and poorly understood toxicity. Lamivudine may cause or exacerbate peripheral neuropathies [18]. Peripheral neuropathy is an established adverse reaction with tenofovir [19]. Zidovudine has lower incidences of peripheral neuropathy (about 5%) [20,21]. In general, NRTIs with higher ATN incidence rates are being prescribed less frequently [22]. However, all NRTIs cause ATN in at least some patients, and we currently lack effective treatment options to help these patients [23]. This lack of effective treatment options is at least in part due to a lacking understanding of ATN and HIV-SN.

**Table 1:** Currently or previously approved NRTI drugs, showing each with their common abbreviation, clinical indication(s), year of initial approval, current status in the United States, major adverse effects, percentage incidence of peripheral neuropathy in drug-treated patients, and strength of inhibition of mitochondrial DNA polymerase-y (3TC, FTC, ABC, and TDF all have equally negligible effects on poly-y).

NRTI	Abbreviation	Clinical Indication(s)	Year Approved	Current Status (US)	Major Adverse Effects	Neuropathy Incidence	Rank Order of Strength of Poly-y Inhibition (5 Greatest—1 Least)
Zidovudine [20]	ZDV, AZT	HIV-1 treatment, postexposure prophylaxis, perinatal transmission prevention	1987	Approved	Hematologic toxicity, myopathy, lactic acidosis	5% or greater	2 [24]
Didanosine [25]	ddI	HIV-1 treatment	1991	Discontinued	Pancreatitis, lactic acidosis, peripheral neuropathy	15–25%	4 [24]
Stavudine [26]	d4T	HIV-1 treatment	1994	Discontinued	Pancreatitis, lactic acidosis, peripheral neuropathy	15–30%	3 [24]
Zalcitabine [27]	ddC	HIV-1 treatment	1992	Withdrawn (2006)	Pancreatitis, lactic acidosis, severe neuropathy, hepatic failure	30–100%	5 [24]
Lamivudine [28]	3TC	HIV-1 treatment, postexposure prophylaxis, HBV treatment	1995	Approved	HBV exacerbation, resistance, pancreatitis, lactic acidosis, neuropathy	≤15%	1 [24]
Emtricitabine [29]	FTC	HIV-1 treatment, postexposure prophylaxis	2003	Approved	HBV acute exacerbation, lactic acidosis	≤4%	-
Abacavir [30]	ABC	HIV-1 treatment	1998	Approved	Severe allergic reaction	N/A	1 [24]
Tenofovir disoproxil fumarate [31]	TDF	HBV infection and prophylaxis, HIV-1 infection and postexposure prophylaxis	2001	Approved	Decreased BMD, osteomalacia, renal toxicity, lactic acidosis	5%	1 [24]

## 3. Peripheral Neuropathy and the Structure of the Peripheral Nervous System

The peripheral nervous system (PNS) primarily consists of nerves that relay signals to and from the central nervous system (CNS) [32]. Of particular interest is the somatic nervous system (SNS) of the PNS, which is responsible for voluntary muscle control (such as skeletal muscle movement) and somatic sensation [32]. The SNS is further subdivided into the sensory division—comprised of afferent neurons that transmit signals (such as pain) from sensory receptors or organs to the CNS and the motor division—composed of efferent neurons that carry signals from the CNS to effectors (like skeletal muscles and organs) [32]. The neuron is the basic unit of the PNS and is comprised of a soma (cell body), dendrites, and an axon [33]. The soma controls cell activity and contains the nucleus and specific organelles for protein and energy production [33]. Dendrites are small branch-like extensions on a neuron that receive chemical signals from other neurons, conduct electrical impulses toward the cell body of the nerve, and transmit electrical impulses through the nervous system to the brain [33]. The axon (i.e., nerve fiber) is a long appendage extending from the cell body that carries information away from the soma [33]. Large axons in the PNS are wrapped in myelin, an insulating sheath that facilitates the rapid conduction of signals along the axon [33]. Groups of neuronal cell bodies, known as ganglia, act as synaptic relay stations between neurons [33]. The dorsal root ganglia, the most common type of sensory ganglia, relay signals from the PNS to the CNS [33]. The sensory ganglia are also found in cranial nerves, which emerge from the basal regions of the brain and interact with the sensory organs of the head and neck [33].

Glial cells support neurons of the PNS and play an important role in nerve injury [34]. One type of glial cell, the Schwann cell (SC), may be either myelinating or non-myelinating [34,35]. Myelinating SCs produce myelin with which they enrobe large axons. Non-myelinating SCs, also called Remak Schwann cells, surround multiple small axons without ensheathing them in myelin [34]. SCs play a critical role in neuronal damage recovery and repair [34,35]. Damage to a peripheral nerve triggers Wallerian degeneration, during which SCs undergo morphological and biological change, enabling endogenous axonal regeneration [34,35,36]. SCs also recruit macrophages through chemokine release, which contributes to recovery by debris removal [35,36]. Satellite glial cells (SGCs), another type of glial cell, are located in the sensory ganglia of the PNS and tightly sheath neuronal cell bodies [37]. In sensory ganglia, SGCs form a sheath surrounding each neuron, the number increasing in relation to the neuron’s volume and surface area [37]. Nerve injury significantly alters SGCs structurally and functionally [38]; SGCs multiply rapidly following nerve injury, express high levels of neurotrophins and cytokines, and undergo the significant activation of MAPK proteins [38]. SGCs may participate in the repair process and may have the ability to replenish damaged neurons [38].

Neuronal and glial cell processes are crucial in preventing and recovering from ATN. Both disease- and xenobiotic-induced neuropathy likely involve neurons and glial cells. Further research characterizing the interplay between neurons and glial cells will elucidate the underlying repair mechanisms and could help develop strategies to prevent neuropathy. Of particular interest is our evolving understanding of autophagy.

Autophagy plays an important role in neuronal homeostasis. Autophagy is a highly regulated intracellular process for the degradation of cytosolic organelles and proteins [39]. This process is particularly important for post-mitotic terminally differentiated cells such as neurons to facilitate the removal of aggregated, aged, or defective organelles and proteins in order to improve the longevity of the cells [40]. Autophagy plays a critical role in neuronal homeostasis, which starts as early as the differentiation of neurons from neuronal stem cells (NSCs). During a study on the murine embryonic olfactory bulb, it was observed that there is a progressive accumulation of autophagy-associated proteins, including Beclin1, AMBRA1, ATG7, and LC3, which drive the differentiation from the NSCs [41]. Another study in human placenta-derived mesenchymal cells reported decreased differentiation when treated with the autophagy inhibitor chloroquine (CQ) for 7 days, suggesting a direct relationship between autophagy and neuronal differentiation [42]. Upon development, the migration of neurons is also under the influence of autophagy. A study concluded that the inhibition of autophagy in cultured neurons reduced the lysosomal degradation of focal adhesion proteins and reduced the neuron migration rate [41]. Neuronal hypertrophy, or the overgrowth of neurites (axons and dendrites), is unfavorable and linked to neuronal disorders and aging, which slows the excitability and motor functions. A study in Atg7- and Atg9-deficient mice showed neurite outgrowth [41]. Another study inhibited autophagy and saw increased branching and neurite complexity. The overgrowth was seen to be reversed with rapamycin treatment in Pten-deficient mice, indicating the importance of autophagy in curbing hypertrophy and maintaining neuronal integrity [43]. Autophagy also regulates synaptic homeostasis and has been shown to play important roles in synaptogenesis and function [44]. The induction of autophagy is associated with controlling neuronal hyperexcitation; for instance, autophagy-deficient cultured hippocampal neurons inhibited the elimination of excessive dendritic postsynaptic protrusions (spines) [45]. Another study suggests that ATG5 regulates the cAMP–PKA–CREB1 axis in neurons, controlling neuronal excitability [46]. Apart from nerve connections, it was also observed in a study that ATG7 deficiency led to structural and functional defects within neuromuscular junctions, and the deleterious effects can be reversed by rescuing Atg7 expression [47]. Autophagy in axons is also responsible for pre-synaptic neurotransmission as well as postsynaptic receptor degradation [48]. Taken together, these reports suggest an indispensable role for autophagy in neuronal development, homeostasis, and function.

## 4. HIV-Associated Neuropathy

Up to half of PLWH experience peripheral neuropathy (HIV-SN) [8]. As introduced previously, the pathophysiology of this peripheral neuropathy is multifaceted and likely involves both direct (e.g., viral toxicity) and indirect (e.g., NRTI toxicity, inflammation, mitochondrial toxicity, or toxicity of treatment for tuberculosis) mechanisms [49]. The general pathophysiology of HIV-induced neuropathy has been reviewed elsewhere [23,49,50,51]. Briefly, viral proteins, including Tat, Vpr, and gp120, are associated with neurotoxicity [52]. However, given that clinical NRTI toxicity necessitates the presence of HIV, we will briefly examine the preclinical evidence for HIV-SN. Animal models of HIV-SN have been difficult to develop, as HIV is species-specific [53]. Nevertheless, animal models of HIV-SN have been developed, as summarized in Table 2.

In general, animals exposed to HIV particles, especially gp120 [58,59], develop symptoms that resemble peripheral neuropathy [54,56,62]. These data support the hypothesis that the HIV virus itself is neurotoxic and may cause HIV-SN. Interestingly, animal models of HIV-SN have demonstrated that the administration of NRTIs has synergistically resulted in an exacerbated HIV-SN phenotype [55,57]. Consistent with these data, animal models of ATN have been developed, which have been used to demonstrate that NRTIs are neurotoxic.

## 5. NRTI-Induced Neuropathy

NRTIs are known to affect individual components of the PNS, resulting in neuropathies. During a study on ddC, it was observed that rabbits receiving a high dose of the drug developed mitochondrial Schwannopathy, impairing SC metabolism and a reduction in the levels of Po mRNA. Another independent study on ddC-treated rats revealed dose-dependent axonal neuropathy, in which mitochondria showed abnormalities [13]. Another study reports the enhanced expression of the pain receptor P2Y_12_ in gp120-injected ddC-treated rat DRG [63].

Preclinical models of NRTI-induced neuropathy have successfully established the neurotoxicity of these agents [11,64], as summarized in Table 3. In general, the administration of NRTIs, but not other components of HAART [65], causes increased mechanical sensitivity [66,67,68,69]. Preclinical models have been utilized to establish the importance of the mitochondria [70] and JNK signaling pathway [69] to observe ATN phenotypes. HIV- and NRTI-induced peripheral neuropathy animal models help inform and identify possible underlying pathological mechanisms occurring in human patients undergoing NRTI treatment and may shed light on potential treatments. In vitro, in vivo, and clinical data have been utilized to develop hypotheses explaining HIV-SN and ATN. We will briefly discuss the strengths and limitations of current hypotheses, especially the poly-γ hypothesis.

## 6. Mechanisms of NRTI-Induced Peripheral Neuropathy

One of the primary causes of ATN is NRTI-induced mitochondrial dysfunction within neurons [73]. Similar mitochondrial dysfunction has been observed in the context of chemotherapy-induced peripheral neuropathy (CIPN) [74,75], supporting the notion that functional mitochondria are important for neuronal health. The mitochondrion is unique among cellular organelles in that it contains its own DNA (mtDNA), responsible for the coding of 13 key electron transport chain (ETC) polypeptides [24]. Mitochondrial DNA is replicated exclusively by mitochondrial DNA polymerase gamma (poly-γ) [24]. Structurally, NRTIs are riboside analogs lacking a 3′ hydroxyl group and are thus able to be transported by natural nucleoside transporters into the cell, where they are then successively phosphorylated to the active triphosphate form [76]. The NRTI will then be incorporated by RT into the growing viral DNA strand, but, due to the lack of the 3′ OH group, a new 3′–5′ phosphodiester bond cannot be formed, resulting in chain termination. Although the above is desirable in the case of RT, NRTIs also have affinity for poly-γ at therapeutic doses, leading to inhibition and thus mtDNA depletion [77,78]. A hypothesis for ATN is the “DNA poly-γ hypothesis” [79], which holds that through their mechanism of action, NRTIs inhibit mitochondrial DNA polymerase-γ (poly-γ), which causes the depletion of mitochondrial DNA (mtDNA), leading to the depletion of key mitochondrial proteins, particularly for the ETC [73]. In turn, this disrupts the oxidative phosphorylation process, resulting in decreased ATP production and electron leakage from the ETC assembly [79]. Due to the leakage of electrons from the ETC, reactive oxygen species (ROS) are generated and released in higher quantities than under normal conditions (although the mitochondrion produces ROS naturally, it can normally counteract these with intracellular antioxidants) [80]. The drastic increase in free ROS can then cause significant damage to mitochondrial lipids, proteins, and mtDNA itself, triggering further oxidative damage [79].

## 7. The Poly-γ Hypothesis and Its Limitations

Significant differences have been observed in the affinity of NRTIs for poly-γ (Table 1). A higher affinity for poly-γ will result in greater enzyme inhibition and mtDNA depletion [77]. The relative affinity of the NRTIs is ddC >> ddI > d4T ≥ AZT >>> TDF = 3TC = FTC = ABC [24].

There are several potential mechanisms by which NRTIs exert their effects under the poly-γ hypothesis. The first possible mechanism is simply the direct competitive inhibition of poly-γ by the activated triphosphate form of the NRTI [79]. The NRTI triphosphate will compete with endogenous deoxynucleotide triphosphates (dNTPs), preventing their incorporation into the growing mtDNA strand and thus inhibiting mtDNA replication [81]. The second possible mechanism is that the NRTI is incorporated into the mtDNA strand [79]. NRTIs have a high enough affinity for poly-γ for the enzyme to incorporate them into the growing mtDNA strand [77]. They will then cause chain termination and halt mtDNA replication [79]. Mechanism 3 involves the alteration of the proofreading capability of poly-γ by the NRTI [79]. The monophosphate forms of some NRTIs, such as AZT, have been shown to accumulate to millimolar concentrations within cells [82,83]. The monophosphorylated NRTI is able to inhibit the exonuclease activity of poly-γ, preventing the enzyme from removing incorrectly paired bases [79]. Thus, with greatly lowered fidelity, mtDNA depletion and mutation will occur [79]. A fourth possible mechanism involves the incorporation of the NRTI into mtDNA, but, crucially, the persistence of the NRTI within mtDNA [79]. As in mechanism 2, the NRTI is incorporated into the mtDNA strand, but instead of simply causing chain termination, the NRTI persists within a growing mtDNA strand [79]. This is due to the inability of the exonuclease portion of poly-γ to efficiently bind and remove the NRTI [79].

Despite the mechanisms proposed by the poly-γ hypothesis, it cannot explain all aspects of NRTI-induced neuropathy. For example, mitochondrial dysfunction has been shown to still occur during treatment with NRTIs that have extremely low affinity for poly-γ. These NRTIs include TDF, which has low affinity for the enzyme, has been shown in numerous studies to exhibit renal toxicity as well as some degree of peripheral neuropathy, which has been further shown to be a result of mitochondrial dysfunction in renal tubular cells [84,85,86]. Additionally, the incidence of neuropathy with 3TC is up to 15%, despite the low affinity for poly-γ [77]. Therefore, the correlation between mtDNA depletion and mitochondrial dysfunction is weak, which directly contradicts the poly-γ hypothesis [24].

## 8. mtDNA Mutation and Oxidative Stress

Beyond the poly-γ hypothesis, an additional hypothesis proposes that the induction of oxidative stress on the mitochondria and the mutation of mtDNA during NRTI exposure results in neuropathy. As the mtDNA copy number decreases with the inhibition of poly-γ, there will be a corresponding decrease in the number of OXPHOS proteins encoded by mtDNA [79]. With the OXPHOS process inhibited, there will be an increase in energy loss from the ETC (through the loss of ATP), as well as significant electron leakage [79]. The increased release of electrons from the ETC will lead to an increase in free radicals within the mitochondrion [80]. Free radicals have the potential to cause significant oxidative damage to many cellular components, including lipids, proteins, and mtDNA itself [77,79]. Several studies have demonstrated mtDNA mutation with NRTI exposure both in vitro and in vivo, with one study demonstrating mtDNA mutation in 5 out of 26 patients treated with AZT for 24 months [87].

The process described above, in which an imbalance between ROS and cellular antioxidants occurs, is also known as oxidative stress [88]. As mitochondria are the sites of OXPHOS, they have the capacity to produce large quantities of ROS, such as superoxide and hydrogen peroxide [88]. Under normal conditions, ROS are managed by endogenous cellular antioxidants, such as glutathione, as well as enzymes designed to eliminate ROS [88]. One important function of the mitochondrion is to concentrate iron for incorporation into heme-containing enzymes, such as the cytochrome enzymes, and non-heme-containing enzymes, including aconitase [89]. Aconitase, which contains an Fe–S bond, is a crucial component of the Krebs cycle and is essential for proper ATP generation through the ETC [79]. ROS can cause the release of Fe(II) from aconitase, which can then bind to mtDNA directly, providing a specific site for oxidation of the mtDNA template [90]. Thus, the significant ROS release under NRTI-induced oxidative stress can lead to significant damage to and the mutation of mtDNA [79]. One study showed that the AZT treatment of lymphoid tissue in vitro led to a 60% decrease in glutathione concentration, decreased ATP production, and the loss of integrity of mtDNA [79], while another demonstrated the same effect in skeletal muscle [91]. Furthermore, an in vivo study in primates demonstrated that chronic d4T exposure led to a decrease in OXPHOS proteins, specifically those encoded by mtDNA [92].

## 9. NRTI-Induced Autophagy Inhibition in Peripheral Nerves as a Novel Toxicological Mechanism

Nerve damage induced by anti-HIV NRTIs impacts the axons of the sensory and motor nerves [93]. As discussed earlier, autophagy plays an important role in the neuronal lifecycle. Furthermore, since neurons do not undergo a cell cycle, autophagy would serve as a recovery mechanism facilitating the clearance of damaged proteins. Several studies have reported an increase in autophagy-related proteins, including Beclin-1, ATG5, ATG7, and LC3II, in neurons and Schwann cells following nerve injury [94]. In Schwann cells, the breakdown of myelin proteins MPZ and MBP was shown to be mediated by autophagy and necessary for remyelination and recovery from nerve injury [95]. Another study pointed out that following a nerve injury, in the absence of autophagy induction, immune cells such as mast cells invaded the injured area, which could be associated with persistent neuropathic pain [96]. A study on sciatic nerve crush injury in rats has reported that the induction of autophagy in the sciatic nerve with rapamycin augmented axon and Schwann cell recovery and decreased cell death [97]. A similar study further showed that mTOR inhibition with high doses of rapamycin (6 mg/kg) had similar effects to the standard-of-care gabapentin, with an improvement in nerve conduction velocities and axon diameter [98]. The targeted inhibition of the autophagic protein ATG14 by miR-195 inhibited autophagy and increased neuroinflammation and neuropathic pain in microglia [99]. Together, these studies suggest that autophagy is important for neuronal health and that dysfunctional autophagic pathways cause neuroinflammation and neuropathic pain. It is possible that neuropathy-causing xenobiotics cause neuropathy through the inhibition of autophagy.

Several recent studies have also shown autophagy improves recovery from CIPN. For instance, the chronic administration of rapamycin reduced cisplatin-induced toxicity in mice as well as in cultured cortical neurons [100]. A study focusing on neuropathy induced by the anticancer agent bortezomib reported a significant decrease in acute analgesia induced by chemotherapeutics when co-administered with mTOR inhibitors including rapamycin and everolimus [101]. Paclitaxel treatment induces autophagy dysfunction in DRG neurons, leading to the accumulation of damaged mitochondria and, subsequently, peripheral neuropathy. The condition was improved upon AICAR administration, with an increased paw withdrawal threshold and licking latency, whereas autophagy inhibition by 3-MA failed to restore nerve fiber loss [102]. These observations collectively point toward the fact that the dysregulation of neuronal autophagy can be detrimental for nerve cell homeostasis and delay recovery from chemotherapeutic toxicities. It is possible that NRTIs cause neuropathy and impact neuronal health through similar mechanisms.

Several recent studies suggest that NRTIs can affect cellular autophagy processes. For instance, a study on the myocyte cell line C2C12 reported that AZT inhibited late autophagy stages, as seen from an increase in autophagosome punctae; this is consistent with a decrease in autophagic flux at therapeutic C_max_. Subsequently, an increase in hyperpolarized mitochondria and ROS generation was also observed at levels similar to those produced by pharmacological autophagy inhibition by 3-MA and nocodazole–vinblastine [103]. Another study on AZT in rats reported similar observations, with an increased number of autophagosomes, as seen from LC3-II as a marker, as well as a reduction in autophagy activation, as seen from the activation of mTOR and the inhibition of AMPK and ULK1 [104]. A report summarizing the neurotoxic effects of NRTIs suggests that combinations of AZT/indinavir, AZT/abacavir, and 3TC/indinavir showed an amyloidogenic effect, indicating the increased production and accumulation of amyloid-β peptide linked to neurodegeneration [105]. Furthermore, reports of amyloid-β being degraded by autophagic pathways suggest that AZT and 3TC affecting autophagy processes is responsible for neurotoxic occurrences [106]. Although amyloid-β is associated with the central nervous system, NRTIs could similarly inhibit autophagic degradation, thereby causing the accumulation of the neurotoxic oligomers linked to peripheral neuropathy. Another study reports that a combination ARV treatment reduced neuronal ATP, which led to the activation of AMPK, yet it failed to restore homeostatic neuronal ATP levels; however, rapamycin exposure averted this ATP deficit [107]. Furthermore, as discussed earlier, HIV has the potential to induce neuropathy. Prolonged NRTI exposure can inhibit autophagy, exacerbating HIV-induced neuropathic pain. Although studies indicate that NRTIs, especially AZT, have a detrimental effect on autophagy, leading to neuropathies, the mechanisms underlying the impairment of autophagy by NRTIs remain unclear.

## 10. Ribonucleotide Pool Depletion

Another possible mechanism beyond the poly-γ hypothesis involves the depletion of deoxyribonucleotide (dRN) and ribonucleotide (RN) pools within the cell [81]. Since NRTIs are riboside analogs, they likely compete with endogenous RNs and dRNs, leading to the depletion of endogenous ribosides and, in turn, mitochondrial dysfunction [81]. A study conducted in 2014 examined the RN and dRN pool size in 75 patients in the setting of NRTI treatment [81]. Twenty-five patients were negative controls (uninfected), 25 were positive controls (HIV infection and NRTI use without mitochondrial toxicity), and 25 were “cases” with both NRTI treatment and mitochondrial toxicity [81]. There was a significant decrease in RN pool size in all three groups, with the positive controls having the highest count [81]. The authors also investigated the mtDNA copy number, a crucial component of the poly-γ hypothesis [81]. However, instead of observing mtDNA depletion in the cases, upregulation of the mtDNA copy number was observed [81]. A similar upregulation of mtDNA has since been observed in several similar studies [73,108,109]. Interestingly, select transporters (CNT1, ENT1, ENT2, OCT1, OCT2, OAT1, OAT2) and metabolic enzymes (TK1, TK2, dCK) were upregulated in the cases [81].

## 11. The Role of Drug Transporters and Metabolism in NRTI-Induced Toxicities

Drug transporters facilitate the movement of drugs across the cell membrane. The expression and localization of these transporters impact the bioavailability and systemic distribution of various drugs. Two major superfamilies of drug transporters exist—the ATP-binding cassette (ABC) and solute carrier (SLC) superfamilies. The ABC superfamily, which contains three subfamilies of efflux transporters, including P-glycoprotein (P-gp; ABCB1), multidrug resistance-associated protein 2 (MRP2; ABCC2), and the breast cancer resistance protein (BCRP; ABCG2) [110], generally reduces the accumulation of substrates within cells or organs, which may reduce oral bioavailability [111] or penetration across the blood brain barrier [112]. The SLC superfamily is a family of more than 300 membrane-bound proteins that facilitate the transport of a wide array of compounds and accomplish numerous physiological processes, including the transport of xenobiotics [113].

Consistent with the notion that chemotherapeutics are directly neurotoxic, and that neuropathy is a function of accumulation within neurons, both ABC [114] and SLC transporters [114,115] have been linked to CIPN. The ability for efflux transport by ABC transporters to reduce accumulation within cancer cells and contribute to drug resistance is well-established [116]. Thus, it is tempting to speculate that the inhibition of ABC transporters could improve outcomes in the treatment of cancer. However, it is possible that the pharmacologic inhibition or genetic deficiency of ABC transporters could result in greater accumulation within the PNS and worsen CIPN. Consistent with this hypothesis, a clinical trial found that CIPN with paclitaxel was worsened in the presence of valspodar, a strong P-gp inhibitor, despite unchanged plasma concentrations [117]. Supporting the role of P-gp in this effect, genetic variants in efflux transporters expressed in the PNS are associated with a greater risk of CIPN with paclitaxel, docetaxel, and vincristine [114], and preclinical evidence suggests that P-gp inhibition causes the accumulation of chemotherapeutics within cultured neurons [118]. Collectively, these data suggest that efflux transporters within the PNS are protective against CIPN. Similarly, there is growing evidence that the accumulation of chemotherapeutics within healthy tissues is mediated by select SLC transporters.

SLC transporters may transport chemotherapeutics into healthy tissues and cause toxicity [115]. For example, OCT2 (SLC22A2) and OCT3 (SLC22A3) have been characterized as critical mediators of cisplatin-induced nephrotoxicity [119,120] and doxorubicin-induced cardiotoxicity [121], respectively. In the context of CIPN, preclinical evidence suggests that SLC transporters play a critical role in CIPN mediated by paclitaxel (OATP1B1/3 (SLCO1B1/3)) [122], vincristine (OATP1B3 (SLCO1B3)) [123], and oxaliplatin (OCT2 (SLC22A2)) [124,125]. Both the genetic deficiency and pharmacologic inhibition of these transporters resulted in protection from neuropathy and the reduced accumulation of the putative agent into the DRG. Given the role of drug transporters in the ability of NRTIs to cross cellular membranes, it is possible that similar mechanisms contribute to ATN.

As nucleoside analogs, NRTIs are transported by various nucleoside transport mechanisms into the cell as well as into the mitochondria. Although endogenous nucleosides and certain analogs are generally hydrophilic, NRTIs lack crucial hydrophilic groups, such as 2′, 3′, and/or 5′ hydroxyl groups, and cannot cross cell membranes through diffusion [76]. Instead, they largely require drug transporter proteins to facilitate cellular entry [76]. The three major gene families that encode the relevant transport proteins are as follows: SLC22 encodes organic anion transporter (OAT) proteins and organic cation transporter (OCT) proteins, SLC28 encodes concentrative nucleotide transporters (CNTs), and SLC29 encodes equilibrative nucleotide transporters (ENTs) [76,79]. As summarized in Table 4, these transporters account for the vast majority of occurrences of NRTI cellular uptake based on both in vitro and in vivo studies [76].

Nucleoside analogs have been developed for the treatment of viral infections and for the treatment of cancer [155]. Despite the well-established notion that SLC transporters are responsible for their cellular uptake and that tissue-specific toxicity may be due to tissue-specific SLC expression [155,156], there is a paucity of direct evidence that these transporters are involved in toxicities caused by nucleoside analogs. Although the anticancer nucleoside analogs nelarabine (ara-G) [157] and cytarabine (ara-C) [158] both cause neurotoxicity at high doses and are transported by ENT transporters [159,160], a causal relationship remains unclear. As discussed earlier, tenofovir renal toxicity is a result of mitochondrial dysfunction in renal tubular cells [84,85,86]. Although the deoxynucleotide carrier (DNC) was originally believed to transport antiretroviral drugs across the mitochondrial membrane [161], this was later questioned when DNC did not contribute to NRTI-induced mtDNA depletion [155,162]. After this, tenofovir was established as a substrate of OAT1 (SLC22A6) [150], OAT3 (SLC22A8) [151], and MRP4 (ABCC4) [153]. Supporting the role of SLC transporters in NRTI-mediated toxicities, a deficiency of OAT1 protected against tenofovir-induced kidney injury, whereas a deficiency of MRP4 exacerbated kidney injury [84]. Despite this promising finding over a decade ago, additional direct evidence linking the SLC transport of NRTIs to tissue-specific toxicities remains lacking. Interestingly, 3TC, which causes neuropathy more frequently than its affinity for poly-γ would suggest, is a substrate of OCT2, which has been implicated in OCT2-mediated oxaliplatin neuropathy [124,125]; however, the role of drug transport in 3TC-induced neuropathy has not been studied. Altogether, it is tempting to speculate that the observed disconnect between the poly-γ hypothesis and the toxicity profile of NRTIs can be partially attributed to their affinity for select drug transporters and the expression patterns of those transporters. However, this area of research has not been explored thoroughly.

Based on the described seminal work on the role of ABC and SLC transporters in CIPN, the importance of nucleoside transporters for NRTIs, and the established association between NRTIs and ATN, we hypothesize that nucleoside transporters play a pivotal role in the accumulation of NRTIs within the PNS and the associated ATN phenotype. Our group has been working on equilibrative nucleoside transporter 3 (ENT3; SLC29A3)—an intracellular lysosomal transporter. ENT3, a high-capacity adenosine transporter, utilizes a pH-sensing mechanism, enabling the lysosomal export of nucleoside substrates (i.e., from the acidic lysosomal compartment to the cytosol) [163,164]. Interestingly, apart from endogenous natural nucleosides (like adenosine), we reported the transport of nucleoside analogs including NRTIs (AZT, 3TC, d4T, ddC, and ddI) by ENT3 [165]. Further, in a separate study, we reported that ENT3 loss leads to the intralysosomal accumulation of adenosine and hampers autophagy by dysregulating the AMPK–mTOR–ULK signaling axis [166]. The data from our current studies suggest NRTIs significantly impair AMPK activation and mTOR suppression, which affects autophagy, as observed from p62 degradation. Taken together, these studies hint toward the potential competitive inhibition of the lysosomal export of adenosine by NRTIs. Thus, NRTIs may impair autophagic processes, similar to what occurs under ENT3-deficient conditions. NRTI-induced peripheral neuropathy could therefore be closely associated with compromised lysosomal ENT3 functions rather than other dysfunctional organelles. We believe future studies will guide us in understanding NRTI-induced neuropathies and the potential role of drug transporters, including ENT3.

The metabolic conversion of the prodrug NRTI to its active form could also play a crucial role in determining toxicity. Once inside the cell, the NRTI is phosphorylated by host cell enzymes to its active triphosphate form before it is able to act on HIV-RT (with the exception of TDF, which is a nucleotide reverse transcriptase inhibitor and is active in its diphosphate form, TFV-DP) [24,76]. NRTIs, as purine or pyrimidine analogs, are metabolized by different enzymes based on their structural differences. For example, AZT, which contains thymine as its base, is metabolized in a stepwise manner by thymidine kinase (TK1 or TK2) to AZT-MP, followed by dTMPK to AZT-DP, and finally by NDPK to AZT-TP [79]. The process is similar among NRTIs. The differential expression of phosphorylation enzymes may play a role in NRTI-induced toxicity. Continuing with the example of AZT metabolism, TK1 is expressed primarily in the cytoplasm, while TK2 is expressed primarily in the mitochondria [79]. Additionally, TK1 is much more efficient in its conversion of AZT to AZT-MP compared to TK2 [79]. It has been shown that tissues with higher ratios of TK2/TK1 show more signs of mitochondrial dysfunction [79]. Further study is crucial to further the understanding of how the transport and metabolism of NRTIs may be an essential factor in mitochondrial dysfunction and ATN.

## 12. Conclusions and Future Directions

NRTIs remain the backbone of contemporary HAART regimens due to their affordability, availability, efficacy, and lower susceptibility to resistance, despite the potential for serious toxicities, including neuropathy. The approach of transitioning to alternative therapy regimens (NRTI replacement) results in worse outcomes, suggesting that NRTIs are an essential component for effective treatment [167,168]. Despite this, to date, there are no effective therapies to treat or prevent HIV-SN and ATN.

Here, we have reviewed both previously described mechanisms, including the poly-γ hypothesis, mtDNA mutation and oxidative stress, ribonucleotide pool depletion, and the potential contribution of novel mechanisms, including NRTI-induced autophagy inhibition and the role of drug transporters and drug metabolizing enzymes. As reviewed, the pathogenesis of ATN remains poorly understood and cannot be fully explained by any single hypothesis or mechanism, which limits our ability to develop effective treatments and mitigation strategies. Accumulating evidence suggests that the poly-γ hypothesis cannot fully explain ATN; this notion is supported by evidence for the role of additional mechanisms, including RN/dRN pool depletion, mtDNA mutation, or autophagy inhibition. In particular, emerging evidence suggests that drug transporters may play a crucial role in the development of ATN, both through their role in the cellular accumulation of NRTIs and downstream development of ATN and by their regulation of cellular homeostasis (e.g., regulation of autophagy by ENT3). Based on this evidence, we believe that characterizing the role of drug transporters in the disposition of NRTIs is crucial to understanding their toxicity profile and the development of strategies to prevent these toxicities. This thesis is supported by elegant studies demonstrating the role of OAT1 and MRP4 in renal toxicity mediated by TDF [84]. Our lab is applying a similar approach to understanding the role of drug transporters, particularly nucleoside transporters, in ATN. These and complementary studies will elucidate the mechanisms underlying ATN by identifying the specific cells involved, characterizing the intracellular pharmacokinetics of NRTIs, and determining the role of organelle toxicity, which, collectively, will assist in the development of strategies to predict, prevent, or treat this dose-limiting toxicity.

## Figures and Tables

**Table 2:** Summary of animal models developed for studying HIV-SN.

Animal Model	Observation	Reference
C57Bl6/J iTAT transgenic mice capable of producing the HIV TAT protein upon induction with DOX.	Induction of TAT-led motor and sensory neuropathy along with a reduction in the expression of proteins involved in the electron transport chain and an increase in proteins associated with mitochondrial fission.	[54]
B6/SJL GFAP-gp120 transgenic mice	Mice producing the HIV protein gp120 showed distal axonal degeneration at 12–15 months of age, which was exacerbated after ddI administration.	[55]
B6 mice infected with the LP-BM5 virus	Mice infected with the murine equivalent of HIV (LP-BM5 virus) were more sensitive to mechanical and heat stimuli post 5 weeks of infection, which was associated with the loss of the intraepidermal nerve fibers visualized through the axonal marker PGP9.5.	[56]
C57BL/6 Tg26 transgenic mice	Tg26 transgenic mice express transgene containing tat, env, rev, nef, vif, vpr, and vpu genes. These proteins significantly lowered mechanical and thermal pain thresholds. HIV-induced neuropathy was further exacerbated by ART treatments.	[57]
Wistar rats	Rats perineurally exposed to the HIV protein gp120 developed persistent mechanical hypersensitivity.	[58,59]
Sprague–Dawley × Fisher 344/NHsd F1 rats	Fertilized one-cell eggs from rats were injected with the HIV-1^gag-pol^ clone pEVd1443, which produced an immunocompromised phenotype similar to HIV-1 infection in humans. These rats also showed neurological abnormalities such as circling behavior and hind-limb paralysis at 5–9 months of age.	[60]
Neonatal kittens	Kittens infected with the neurovirulent recombinant molecular clone V1-Ch of the feline immunodeficiency virus (FIV) showed a reduction in the sural nerve axonal count and epidermal nerve fiber density. FIV-infected animals also showed a delayed withdrawal response to thermal stimuli.	[61]
Rhesus macaques	SIVmac251-infected monkeys recapitulated the clinical HIV-DSP phenotype that includes reduced IENF densities, DRG satellitosis, the presence of Nageotte nodules in DRG, neuronophagia, increased numbers of CD68+ macrophages, and abundant viral replication. The SIV-induced neuropathy is exacerbated by the depletion of CD8^+^ lymphocytes.	[62]

**Table 3:** Summary of preclinical NRTI-induced neuropathy models.

Animal Model	Observation	Reference
C57BL/6J mice	Adult mice dosed with FTC developed sensitivity to mechanical stimuli and reduced tail flick time and showed epidermal denervation. PI, INSTI, and NNRTI treatments did not exhibit a neurotoxic response.	[65]
BALB/C mice	Mice treated with ddC showed an increase in the number of intraxonal mitochondria. Furthermore, many neuronal organelles were observed to be unusually large due to hydropic swelling along with distorted architecture.	[70]
CD1 mice	Mice with a single administration of d4T or ddC developed persistent mechanical allodynia, which was mitigated after the inhibition of the JNK pathway.	[69]
Rodents	d4T, ddC, and ddI administration resulted in dose-dependent mechanical and thermal hypersensitivity. This phenotype can be achieved through IP or IV injection, with doses ranging between 10 and 50 mg/kg given for 3 days. An increased myelin sheath thickness and Remak bundle degeneration is evident.	[68]
Wistar rats	Immunohistochemical staining of the cerebellum of rats treated with 3TC revealed degeneration in the form of a distorted granular layer, shrunken Purkinje cells, and increased GFAP staining.	[71]
Sprague–Dawley rats	Rats treated with ddC, ddI, and d4T showed dose-dependent mechanical sensitivity and a non-significant reduction in motor function.	[67]
Wistar rats	d4T treatment increased the sural nerve axonal diameter and decreased the hind paw intraepidermal nerve fiber density. Furthermore, d4T-treated mice showed increased mechanical sensitivity, which was attenuated with analgesics—gabapentin and WIN 55,212-2; behavioral changes: increased thigmotaxis and reduced chances of burrowing.	[66]
Neonatal kittens	In FIV-infected kittens treated with ddI, nerve damage was exacerbated by FIV, as observed from the decreased sural nerve axonal count and epidermal nerve fiber density as well as withdrawal response latency. ddI was also observed to be neurotoxic to cultured DRG neurons and significantly reduced the neurite length.	[61]
Pigtailed macaques	SRV-2-infected monkeys upon treatment with ddC developed signs resembling peripheral neuropathy. The toxicities were observed to be prevalent and more severe with bolus doses rather than prolonged administration.	[72]

**Table 4:** NRTI properties showing the active form of the NRTI, cellular uptake transporters, cellular efflux transporters, and plasma half-life. Recent data detailing the uptake and efflux of zalcitabine is not available, likely due to its withdrawal from the US market. OAT, organic anion transporter; OCT, organic cation transporter; CNT, concentrative nucleoside transporter; ENT, equilibrative nucleoside transporter; MAT, multidrug and toxin extrusion protein; MRP, multidrug resistance-associated protein; BCRP, breast cancer resistance protein; P-gp, P-glycoprotein. CBV, carbovir (the active form of abacavir); TFV, tenofovir.

NRTI	Abbreviation	Active Form	Uptake Transporters	Efflux Transporters	Half-Life (Plasma)	Activating Enzymes
Zidovudine [20]	ZDV, AZT	AZT-TP [76]	OAT1-4, CNT1, CNT3, ENT2 [76]	MRP4, BCRP [126,127]	1–2 h [128]	Thymidine kinase (TKs), thymidylate kinase (dTMPK), and nucleoside diphosphate kinase (NDPK) [79]
Didanosine [25]	ddl	ddl-TP [76]	CNT2, CNT3, ENT1, ENT2 [129,130]	BCRP [127]	2.3 h [131]	ddI-MP—5′nucleotidase, inosine 5′-monophosphate phosphotransferase ddI-DP—adenylate kinase ddI-TP—creatine kinase, phosphoribosyl pyrophosphate synthetase [12]
Stavudine [26]	d4T	d4T-TP [76]	CNT1 [132,133]	BCRP, MRP5 [127,134]	7 h [135]	d4T-MP—thymidine kinase d4T-DP—thymidylate kinase d4T-TP—nucleoside diphosphate kinase [136]
Zalcitabine [27]	ddC	ddC-TP [76]	CNT1 [137], OAT1 [138]	MRP8 [139]	1.1–1.8 h [140]	ddC-MP—deoxycytidine kinase [136]
Lamivudine [28]	3TC	3TC-TP [76]	OCT1, OCT2, CNT1 [141,142]	BCRP [127]	5–7 h [143]	3TC-MP—deoxycytidine kinase 3TC-DP—dCMP kinase 3TC-TP—NDP kinase [144]
Emtricitabine [29]	FTC	FTC-TP [76]	MATE1 [145]	MRP1 [146]	7.4 h [131]	Similar to 3TC [12] Similar to 3TC [12]
Abacavir [30]	ABC	CBV-TP [76]	ENT1 [147]	P-gp, MRP4, BCRP [126,127,148]	1.5 h [149]	ABC-MP- adenosine phosphotransferase CBV-DP- guanylate kinase CBV-TP- diphosphate kinase [12]
Tenofovir disoproxil fumarate [31]	TDF	TFV-DP [76]	OAT1 [150]; OAT3 [151]	P-gp [152], MRP4 [84,153]	17 h [154]	TDF-MP—AMP kinase TDF-DP—AMP kinase [136]

## Abbreviations

HIV-1	Human immunodeficiency virus
HAART	Highly active antiretroviral therapy
NRTI	Nucleoside reverse transcriptase inhibitor
HIV-SN	HIV-related sensory neuropathy
ATN	Antiretroviral toxic neuropathy
3TC	Lamivudine
ABC	Abacavir
AZT	Zidovudine
d4T	Stavudine
ddC	Zalcitabine
ddI	Didanosine
FTC	Emtricitabine
TDF	Tenofovir disoproxil fumarate
CNS	Central nervous system
PNS	Peripheral nervous system
SC	Schwann cells
SGC	Satellite glial cells
